# Biomechanics of the ageing foot and ankle

**DOI:** 10.1186/1757-1146-5-S1-K3

**Published:** 2012-04-10

**Authors:** Hylton B Menz

**Affiliations:** 1 Musculoskeletal Research Centre, La Trobe University, Bundoora, Victoria 3086, Australia

## 

Foot pain affects up to 24% of people aged over 65 years, and is associated with difficulty undertaking activities of daily living, problems with balance and gait, an increased risk of falls, and reduced health-related quality of life. Established risk factors for foot pain in this age-group include female sex, obesity and chronic medical conditions such as osteoarthritis and diabetes. However, given the significant age-related changes in the structure and function of osseous, muscular and soft tissues in the foot, the contribution of lower limb biomechanical factors to the development of foot pain in older people is receiving increased attention in the research literature. This presentation will provide an overview of (i) the epidemiology of foot disorders in older people, (ii) the physiological changes that occur in the ageing foot, (iii) the role of biomechanics in understanding the potential mechanisms underlying the development of foot pain, and (iv) the role of plantar pressure analysis for the assessment and management of foot pain in this age-group.

